# Harnessing Data to Assess Equity of Care by Race, Ethnicity and Language

**DOI:** 10.3390/ijerph13010045

**Published:** 2015-12-22

**Authors:** Amber Gracia, Jorge Cheirif, Juana Veliz, Melissa Reyna, Mara Vecchio, Subhash Aryal

**Affiliations:** 1 Cinco Strategy Group, 937 Kessler Parkway, Dallas, TX 75208, USA; 2 Presbyterian Heart & Vascular Group, 8440 Walnut Hill Lane, #610, Dallas, TX 75231, USA; jorgecheirif@texashealth.org; 3 Texas Health Resources, 612 E. Lamar Blvd, Suite 1000, Arlington, TX 76011, USA; juanaveliz@texashealth.org (J.V.); melissareyna@texashealth.org (M.R.); 4 Texas Health Research & Education Institute, 8400 Walnut Hill Lane, Suite 220, Dallas, TX 75231, USA; maravecchio@texashealth.org; 5 Department of Biostatistics and Epidemiology, University of North Texas Health Science Center, 3500 Camp Bowie Blvd, Fort Worth, TX 76107, USA; subhash.aryal@unthsc.edu

**Keywords:** race, ethnicity, language, populations, disparities

## Abstract

Objective: Determine any disparities in care based on race, ethnicity and language (REaL) by utilizing inpatient (IP) core measures at Texas Health Resources, a large, faith-based, non-profit health care delivery system located in a large, ethnically diverse metropolitan area in Texas. These measures, which were established by the U.S. Centers for Medicare and Medicaid Services (CMS) and The Joint Commission (TJC), help to ensure better accountability for patient outcomes throughout the U.S. health care system. Methods: Sample analysis to understand the architecture of race, ethnicity and language (REaL) variables within the Texas Health clinical database, followed by development of the logic, method and framework for isolating populations and evaluating disparities by race (non-Hispanic White, non-Hispanic Black, Native American/Native Hawaiian/Pacific Islander, Asian and Other); ethnicity (Hispanic and non-Hispanic); and preferred language (English and Spanish). The study is based on use of existing clinical data for four inpatient (IP) core measures: Acute Myocardial Infarction (AMI), Congestive Heart Failure (CHF), Pneumonia (PN) and Surgical Care (SCIP), representing 100% of the sample population. These comprise a high number of cases presenting in our acute care facilities. Findings are based on a sample of clinical data (N = 19,873 cases) for the four inpatient (IP) core measures derived from 13 of Texas Health’s wholly-owned facilities, formulating a set of baseline data. Results: Based on applied method, Texas Health facilities consistently scored high with no discernable race, ethnicity and language (REaL) disparities as evidenced by a low percentage difference to the reference point (non-Hispanic White) on IP core measures, including: AMI (0.3%–1.2%), CHF (0.7%–3.0%), PN (0.5%–3.7%), and SCIP (0–0.7%).

## 1. Introduction

Texas Health Resources is one of the largest health systems in the United States, serving a highly diverse population in North Texas. The Dallas-Fort Worth-Arlington metropolitan statistical area (MSA) alone is home to more than six million people. As reported by the 2010 U.S. Census, the racial makeup in this MSA is predominantly non-Hispanic White at 50.2%, with African-American representing 15.4%, Native American, 0.6%, Asian, 5.9% and Native Hawaiian/Pacific Islander, 0.1%. Approximately 10% are from other races, and 2.4% are from two or more races. Hispanic and Latino of any race represents approximately 27% of the population.

Formed in 1997 with the assets of Fort Worth-based Harris Methodist Health System and Dallas-based Presbyterian Healthcare Resources (Arlington Memorial Hospital joined the Texas Health system later that year), Texas Health became one of the first in North Texas to freely and openly report its quality data to the community—both positive and negative. Comprised of 25 acute-care and short-stay hospitals that are owned, operated or affiliated as joint ventures, this philosophy continues with regards to documenting disparities using race, ethnicity and language (REaL) data parameters.

For the purpose of this discussion, race and ethnicity are defined socially and culturally and, in the case of federal data collection, by legislative and political necessity [1]. As discussed in a report by the U.S. Institute of Medicine [2], the categories of race and ethnicity “are social-political constructs and should not be interpreted as being scientific or anthropologic in nature.” Further, scientific findings provide empirical evidence that there is more genetic variation within than among racial groups; thus, racial categories do not represent major biological distinctions [3]. Despite these flaws in applying the terms race and ethnicity, it nonetheless remains important to use the terms when distinguishing the diversity of the U.S. population.

For standardization purposes, ethnicity is a concept that is distinguished from race. The term ethnicity represents a common ancestral heritage that gives social groups a shared sense of identity. The U.S. Census captures data on a few discrete ethnic groups, including Hispanic, Asian and Native Hawaiian/Pacific Islander. American Indian and Alaska natives also are given the option to indicate a tribal group [3].

Regardless of the semantics, it is especially important to determine if care provided across the U.S. care continuum is equitable, given the current state of the U.S. health care industry, which is rapidly consolidating and shifting from fee-for-service to a value-based system. Where disparities exist, these should be addressed immediately, with any quality and value changes instituted moving forward.

Unfortunately, as few as 14% to 25% of hospitals and care systems in the United States are using race, ethnicity and language (REaL) data at this time to assess variation in quality and health outcomes [4] despite compelling research indicating that racial/ethnic minorities and non-English speakers consistently receive a lower quality of health care compared to non-Hispanic Whites, even with similar health insurance and access to a health care provider [5]. Accurate collection, rigorous analysis and consistent monitoring of REaL data by health systems is needed to correct these disparities while helping to influence policy, focus interventions and ensure equitable and high-quality care for all patients [6].

To help systems monitor their performance, National Quality Improvement Goals have been put into place to begin standardizing performance measures across accredited health care organizations nationwide [7]. In addition, core measures were established based on evidence-based medical research to define baseline (core) care, meaning what is reasonable to expect for every patient with a given diagnosis.

In November 2003, the U.S. Centers for Medicare and Medicaid Services (CMS) and The Joint Commission (TJC) decided to take standardization one-step further, creating one common set of measure specifications. Documented in the Specifications Manual for National Hospital Inpatient Quality Measures [8], this resulted in Appropriateness of Care Scores (ACS) that indicate how often patients received all recommended treatments (measure set) for their clinical condition, with the goal being 100%. The ACS score is determined by quantifying the care received based on a scoring algorithm. If as few as one of the core set measures was missed, the result was a failing score.

In the REaL analysis, Texas Health set out to verify its performance, utilizing existing data, compared against the ACS standard. These are standards that all health care organizations in the U.S. are measured by and are independent of access to care issues. Assessing performance through analysis of retrospective data provides a baseline to monitor performance moving forward.

## 2. Experimental Section

From 1 January 2012 to 31 March 2013, Texas Health conducted its race, ethnicity and language (REaL) retrospective review based on the latest standardized (IP) core measures (appropriateness of care scores). The study sample of clinical data (N = 19,873 cases) was derived from 13 wholly-owned Texas Health facilities.

The four core measures analyzed in (%) samples were Acute Myocardial Infarction (AMI), 13.6%; Congestive Heart Failure (CHF), 20.5%; Pneumonia (PN), 24.8%; and, Surgical Care (SCIP), 41.2%. These were evaluated by race (non-Hispanic White, non-Hispanic Black, Native American/Native Hawaiian/Pacific Islander, Asian and Other), ethnicity (Hispanic and non-Hispanic), and preferred language (English and Spanish).

As defined by the Joint Commission, all of the selected IP core measures are based on four key criteria: (1) research, with evidence-based care demonstrating improved health outcomes; (2) proximity, with care closely connected to the patient outcome; (3) accuracy, ensuring that the care process has been provided; and (4) adverse effects, requiring little or no chance of causing an unintended, adverse consequence. Based on this criteria, these measures have been shown to produce the greatest positive impact on patient outcomes when hospitals demonstrate improvement.

For the baseline period, selected core measures included the following number of indicators in each measure set: 9, AMI; 3, HF; 5, PN; 10, SCIP (reduced to 9 in January 2013).

Results were reported based on the percentage of patients admitted with a specific diagnosis who received the recommended measure set, adhering to a minimum threshold of 30 cases for the process measures (as established by The Joint Commission). The formula for calculating the Appropriateness of Care Score (ACS) is as follows:

$$\frac{\text{Number of cases with given diagnosis receiving recommended care}}{\text{Total number of cases with a given diagnosis}}=\%$$

A case will enter the numerator only if all measures within a given measure set are delivered. If one or more measures within the set were not delivered, then the case would be considered a “missed” case.

During the data collection process, race, ethnicity and language (REaL) data values, which originate from the patient intake process, were adjusted to reflect any changes input by the clinical staff. This adjustment was made based on the assumption that clinical staff make adjustments to the patient record if they find that any of the race, ethnicity and/or language fields contained incorrect information. It is important to note that comparative Hispanic cases with “unknown” language preference were excluded due to an inability to assign cases to a designated language group. The “Other” case population included patients of “unknown” race or “two or more races”.

Differences were measured against the reference group, which is defined as non-Hispanic White, based on majority and historically-advantaged status [9].

### 2.1. Selection and Description of Participants

As mentioned above, the decision was made to use the majority group rate (non-Hispanic White) as a reference point for the analyses. While total population as the reference point would have been valid, it was ruled out as it did not account for population shifts over time. Most favorable population was also was considered valid, but, because it was likely to be a relatively small population within certain diagnoses, there was concern that it could skew the comparison. Therefore, non-Hispanic White was selected due to the potential for the largest data subset. This also enabled comparisons against the care and outcomes of the most advantaged population, and is the dominant racial/ethnic make-up of our care teams.

### 2.2. Technical Information

In this analysis it is important to note inclusion of relative *versus* absolute differences. Absolute measures of disparity are the difference between a group rate and the reference group (non-Hispanic White), whereas relative measures express the disparity as a ratio relative to the reference point (non-Hispanic White), so that the reference point becomes the unit of comparison. During the analysis, both the absolute and relative disparities were assessed, but due to space constraints, only relative measures are included in this presentation.

### 2.3. Statistical Methods

Our primary outcome measure was presence or absence of AMI, CHF, PN and SCIP. As such we calculated the proportion of subjects with the condition present for each diagnosis. We used the large sample approach (Z-test) to test the hypothesis about single population proportion and for comparing the proportion between two independent populations.

The overall alpha = 0.05 was adjusted using the Bonferroni approach to account for multiple testing. Since comparisons were being made for each group (Asian, Native American/Native Hawaiian/Pacific Islander, Hispanic/Latino English and Hispanic/Latino Spanish for a total of four groups) to non-Hispanic Whites, the Bonferroni adjusted *p*-value is (0.05/(2 × 4)) = 0.00625. So, only *p*-values less than 0.00625 were considered to be significant. The Bonferroni adjustment is the most frequently used approach in literature. There are alternative methods, including Tukey, Scheffe’s and false discovery rate [10] to adjust for multiple testing. We performed multiple testing using all Tukey, Scheffe and Bonferroni approaches and arrived at the same conclusion using all three methods. Since the Bonferroni adjustment is the most frequently used approach in literature, we present the Bonferroni-adjusted results. The newer false discovery rate approach can lead to higher false positives so it was not considered suitable for our study.

## 3. Results and Discussion

Initial observations suggest that no major disparities exist in the four prioritized IP core measures based on race, ethnicity, and preferred language within the Texas Health Resources health care delivery system. Details on these findings are represented in the Table 1, Table 2, Table 3 and Table 4 (below).

**Table 1:** Baseline Data Acute Myocardial Infarction (AMI).

Group	Cases (n)	% Cases	AMI_ACS ^1^ (%)	Difference (%)	*p*-Value
Hispanic/Latino	196	7.3	98.47	0.83	0.3752
English	158	80.6	98.73	1.10	0.2480
Spanish	38	19.4	97.37	(0.27)	0.9182
Asian	48	1.8	100.00	2.36	<0.0001
Non-Hispanic Black	227	8.4	96.48	(1.16)	0.3598
Native American/Native Hawaiian/PI	8	0.3	100.00	2.36	<0.0001
Other	145	5.4	97.93	0.29	0.8111
Non-Hispanic White	2074	76.9	97.64		-
Total	2698	100.0	97.66		

**^1^** CMS/TJC Specifications Manual for National Hospital Inpatient Quality Measures 4.0c, 4.1, and 4.2b.

For AMI, noted in Table 1, 196 Hispanic/Latino cases were compared against the non-Hispanic White reference group, with 98.47% of cases receiving all ACS measures. Non-Hispanic Black was equally high at 96.48%. Measurable differences were observed in the Asian cohort (*p* < 0.0001) and Native American/Native Hawaiian/Pacific Islander cohort (*p* < 0.0001). We conducted post-hoc power analysis and observed the power to be above 80% in both cases. The number of cases for Asian and Native American/Native Hawaiian/Pacific Islander is 48 and 8 respectively. Note that the number of cases are relatively small for both these cohorts and we need to use caution while drawing strong inferences about the level of care that these groups received.

In Table 2, for CHF, the findings were similar, with Hispanic/Latino cases meeting all ACS measures at 97.01%; Asian at 96.08%, and non-Hispanic Black at 98.55%.

**Table 2:** Baseline data congestive heart failure (CHF).

Group	Cases (n)	% Cases	CHF_ACS ^1^ (%)	Difference (%)	*p*-Value
Hispanic/Latino	334	8.2	97.01	(0.70)	0.4706
English	239	71.6	97.91	0.20	0.8359
Spanish	95	28.4	94.74	(2.97)	0.1979
Asian	51	1.31	96.08	(1.63)	0.5509
Non-Hispanic Black	690	17.0	98.55	0.84	0.1135
Native American/Native Hawaiian/PI	10	0.25	90.00	(7.71)	0.4167
Other	58	1.4	100.00	2.29	<0.0001
Non-Hispanic White	2923	71.9	97.71		-
Total	4066	100.0	97.79		

**^1^** CMS/TJC Specifications Manual for National Hospital Inpatient Quality Measures 4.0c, 4.1, and 4.2b.

In Table 3, for PN, Hispanic/Latino cases meeting all ACS measures was at 96.94%, with non-Hispanic Black at 97.83%.

**Table 3:** Baseline Data Pneumonia (PN).

Group	Cases (n)	% Cases	PN_ACS ^1^ (%)	Difference (%)	*p*-Value
Hispanic/Latino	359	7.3	96.94	(0.47)	0.6193
English	270	75.2	96.30	(1.11)	0.3461
Spanish	89	24.8	98.88	1.47	0.1989
Asian	55	1.1	100.0	2.60	<0.0001
Non-Hispanic Black	460	9.4	97.83	0.42	0.5614
Native American/Native Hawaiian/PI	11	0.2	100.0	2.60	<0.0001
Other	65	1.3	93.85	(3.56)	0.2342
Non-Hispanic White	3969	80.7	97.40		-
Total	4919	100.0	97.40		

**^1^** CMS/TJC Specifications Manual for National Hospital Inpatient Quality Measures 4.0c, 4.1, and 4.2b.

In Table 4, for SCIP, Hispanic/Latino cases meeting all ACS measures was at 94.85%, with Asian at 95%, Non-Hispanic Black, 93.61% and Other at 94.77%.

**Table 4:** Baseline Data Surgical Care (SCIP).

Group	Cases	% Cases	SCIP_ACS ^1^ (%)	Difference (%)	*p*-Value
Hispanic/Latino	505	6.2	94.85	0.60	0.5556
English	391	77.4	94.88	0.64	0.5798
Spanish	114	22.6	94.74	0.49	0.8169
Asian	140	1.7	95.00	0.75	0.6867
Non-Hispanic Black	657	8.0	93.61	(0.64)	0.5198
Native American/Native Hawaiian/PI	24	0.3	100.00	5.75	<0.0001
Other	153	1.9	94.77	0.52	0.7741
Non-Hispanic White	6711	81.9	94.25		-
Total	8190	100.0	94.27		

**^1^** CMS/TJC Specifications Manual for National Hospital Inpatient Quality Measures 4.0c, 4.1, and 4.2b.

## 4. Discussion

Initial observations suggest that no major discrepancies of the selected IP core measures exist in analyzing race, ethnicity, and preferred language within the Texas Health Resources health care delivery system. However, it should be noted that statistically significant differences for “Other” and “Native American/Native Hawaiian/Pacific Islander” population were observed across the core measure groups, although the sample sizes were relatively small in these groups. The results are statistically significant but due to small sample sizes we should use caution. Measurable differences were observed in the Asian and Native American populations. Again, we would like to urge caution that the number of cases for both of these cohorts were relatively small and even though post-hoc power analysis showed adequate power, strong inferences should not be drawn until additional studies with much larger numbers of cases reproduce similar results.

In light of the recent American Heart Association (AHA) campaign to reduce health care disparities (#123forEquity Pledge to Act) [11], Texas Health Resources already has taken a proactive approach to identifying whether race, ethnicity and language (REaL) disparities exist. The AHA has identified three areas of interest to help eliminate disparities, including the effort to “increase the collection and use of race, ethnicity and language preference data” [12]. Utilizing core measures to determine if disparities exist, this exercise becomes a powerful tool for driving performance improvement.

Reducing disparities can improve the quality and performance of most any health care system that undertakes this exercise, and also has moral and ethical implications. Disparities that exist can’t be addressed if there isn’t a consistent method for utilizing the data. This initial approach taken by Texas Health Resources to review core measure sets and determine if there were “missed” core measures stratified by race, ethnicity and language, provides a method of measurement for variables within its patient population.

### 4.1. Limitations of This Study Include

- Retrospective study
- Due to platform differences, patient data was only available for Texas Health Resources’ wholly-owned facilities
- Cases of unknown race (included in Other cohort)
- Limited size of the Asian and Native American/Native Hawaiian/Pacific Islander cohorts in all four measures analyzed
- Peer group comparisons not available by race or language and only comparable for three measures: AMI, CHF and PN

### 4.2. Recommendations

To further measure equity of care at Texas Health, this study is being expanded to analyze other inpatient core measures and readmissions data. Further analysis of internal data is essential before applying the model externally. To make the data easily available, the research team recommends that a quality dashboard be created, with data compared against baseline measurements on a periodic basis. This control phase of the research can help drive policy decision and initiatives as needed.

## 5. Conclusions

Evaluating performance based on IP measures gave Texas Health Resources a reliable way to determine if any disparities existed in care based on race, ethnicity and language (REaL), and to monitor and correct any noted disparities.
